# Improved visual outcomes after endoscopic endonasal transsphenoidal surgery for pituitary macroadenomas: a single-arm meta-analysis

**DOI:** 10.1007/s00701-026-06967-4

**Published:** 2026-06-24

**Authors:** Khushal Gupta, Moneebah Ashraf, Dorsa Farahnak, Elham Rahmanipour, Maryam Alamdar, Clayton Rawson, David Altschul, Mehrdad Pahlevani, Michael Karsy

**Affiliations:** 1 Department of Neurosurgery, Albert Einstein College of Medicine, New York, NY USA; 2 Central Michigan University College of Medicine, Mount Pleasant, MI USA; 3 University of Turin, Turin, Italy; 4 Immunology Research Center, Mashhad University of Medical Sciences, Mashhad, Iran; 5 Mashhad University of Medical Sciences, Mashhad, Iran; 6 Noorda College of Osteopathic Medicine, Provo, UT USA; 7 Department of Neurosurgery, Jamaica Hospital Medical Center, 8900 Vanwyck Expressway, New York, NY 11418 USA; 8 Department of Neurosurgery, University of Michigan, Michigan Medicine, Ann Arbor, MI USA

**Keywords:** Endoscopic endonasal transsphenoidal surgery, Optic chiasm compression, Pituitary macroadenoma, Visual field, Visual outcome

## Abstract

**Background:**

Visual impairment due to optic chiasm compression is a frequent complication of pituitary macroadenomas and substantially impacts quality of life. Reported postoperative visual field (VF) results after endoscopic endonasal transsphenoidal surgery (EETS) vary across series. We performed a single-arm meta-analysis to quantify postoperative VF outcomes after EETS for pituitary macroadenomas.

**Method:**

PubMed/MEDLINE, Scopus, and Web of Science were searched in accordance with PRISMA through September 2025. Eligible studies were prospective or retrospective clinical series of pituitary macroadenomas (≥ 5 patients) undergoing EETS with extractable postoperative VF outcomes. Pooled proportions for VF improvement, normalization, stability, deterioration, and cerebrospinal fluid (CSF) leak were calculated using random-effects models with Freeman–Tukey double-arcsine transformation. Heterogeneity was assessed using I^2^ and prediction intervals were reported.

**Results:**

Twenty nine studies involving 2,943 patients met criteria. Across studies, the combined rate of postoperative VF improvement was 79% (95% CI: 73–84%), while normalization was achieved in 41% (95% CI: 29–53%). VFs remained unchanged in 19% (95% CI: 14–25%) of patients, and deterioration following surgery was infrequent (1%; 95% CI: 0–1%). The pooled postoperative CSF leak rate was 7% (95% CI: 4–10%).

**Conclusion:**

In pooled single-arm data, EETS for pituitary macroadenomas is associated with high rates of VF improvement and rare postoperative deterioration. Because VF testing and follow-up were heterogeneous across studies, these pooled results should be interpreted as benchmark outcome rates rather than standardized recovery “patterns.” Standardized VF reporting is needed to enable stronger prognostic and time-course analyses.

## Introduction

Pituitary adenomas are classified as benign neoplasms and are among the most frequent and well-characterized primary intracranial tumors, representing approximately 15–20% of all primary intracranial neoplasms, and affecting up to 17% of the population in autopsy and imaging series [18]. Due to proximity to the optic chiasm, pituitary macroadenomas are a predominant cause of compressive chiasmopathy and visual field (VF) loss in adults [16]. Neuro-ophthalmic features are common at presentation and may include bitemporal hemianopia, junctional scotomas, and reduced visual acuity, often serving as the primary indication for tumor excision [16, 41]. The aim of pituitary surgery is to preserve and restore visual function as a crucial factor influencing long-term quality of life [16, 41]. Considering that macroadenomas are the primary source of chiasmal compression and the preoperative VF deficits commonly observed, this review will be limited to macroadenomas.

Endoscopic endonasal transsphenoidal surgery (EETS) has largely replaced microscopic techniques in many centers and is associated with similar or improved rates of gross-total resection and visual improvement, particularly for macroadenomas with suprasellar extension [24, 41]. In the largest meta-analysis of EETS to date, Muskens et al. [41] noted improvement in VF deficits in approximately 80% of patients with 40% achieving complete recovery and around 2% experiencing postoperative deterioration. Subsequent prospective and retrospective series, including more recent endoscopic series, have demonstrated high rates of VF improvement following decompression, although the proportion of complete normalization versus partial improvement varies widely between series [15, 29].

While the overall outcome after EETS is likely favorable, the pattern, timing, and factors influencing VF resolution are not fully elucidated. Clinical and imaging data reveal that baseline VF severity, duration of visual symptoms, tumor size and suprasellar extension, and structural assessments of axonal integrity (e.g., retinal nerve fiber layer thickness) influence the degree and timing of postoperative improvement [16, 29]. Quantitative analyses demonstrate that the majority of VF improvements occur within the initial months following surgery, followed by a plateau phase with minimal late recovery [15, 16, 29]. Previous systematic reviews and meta-analyses have predominantly classified outcomes into “improved” or “not improved,” without providing detailed patterns such as complete normalization, partial improvement, stability, or deterioration [24, 41]. This binary classification limits the ability to provide nuanced, evidence-based counseling to patients with pituitary macroadenomas. Addressing this gap, we conducted a single-arm meta-analysis to quantify postoperative VF outcomes (improvement, normalization, stability, and deterioration) after EETS for pituitary macroadenomas.

## Methods

Literature search and study selection followed PRISMA procedures. PubMed/MEDLINE, Scopus, and Web of Science were searched up to September 2025 for studies reporting VF outcomes following endoscopic endonasal procedures for pituitary tumors. The search strategy incorporated tumor-specific keywords ("pituitary adenoma," "pituitary macroadenoma," "giant pituitary adenoma") alongside surgical approach terminology ("endoscopic transsphenoidal," "endoscopic endonasal," "endoscopic pituitary surgery") and visual outcome descriptors ("visual field," "visual field defect," "visual recovery," "visual outcome") combined with Boolean operators. Inclusion criteria were **prospective or retrospective clinical studies** of pituitary macroadenomas published in English, including **5 or more patients**, with extractable postoperative VF data following an endoscopic endonasal transsphenoidal approach. At title/abstract screening, studies were excluded when they clearly did not meet inclusion criteria (e.g., non-endoscopic or mixed approaches, non-macroadenoma sellar pathology, no postoperative VF outcomes, non-original publications, non-English, or case reports/very small series). Methods of VF assessment were evaluated and studies were included so long as objective assessments were obtained.

**Fig. 1:**
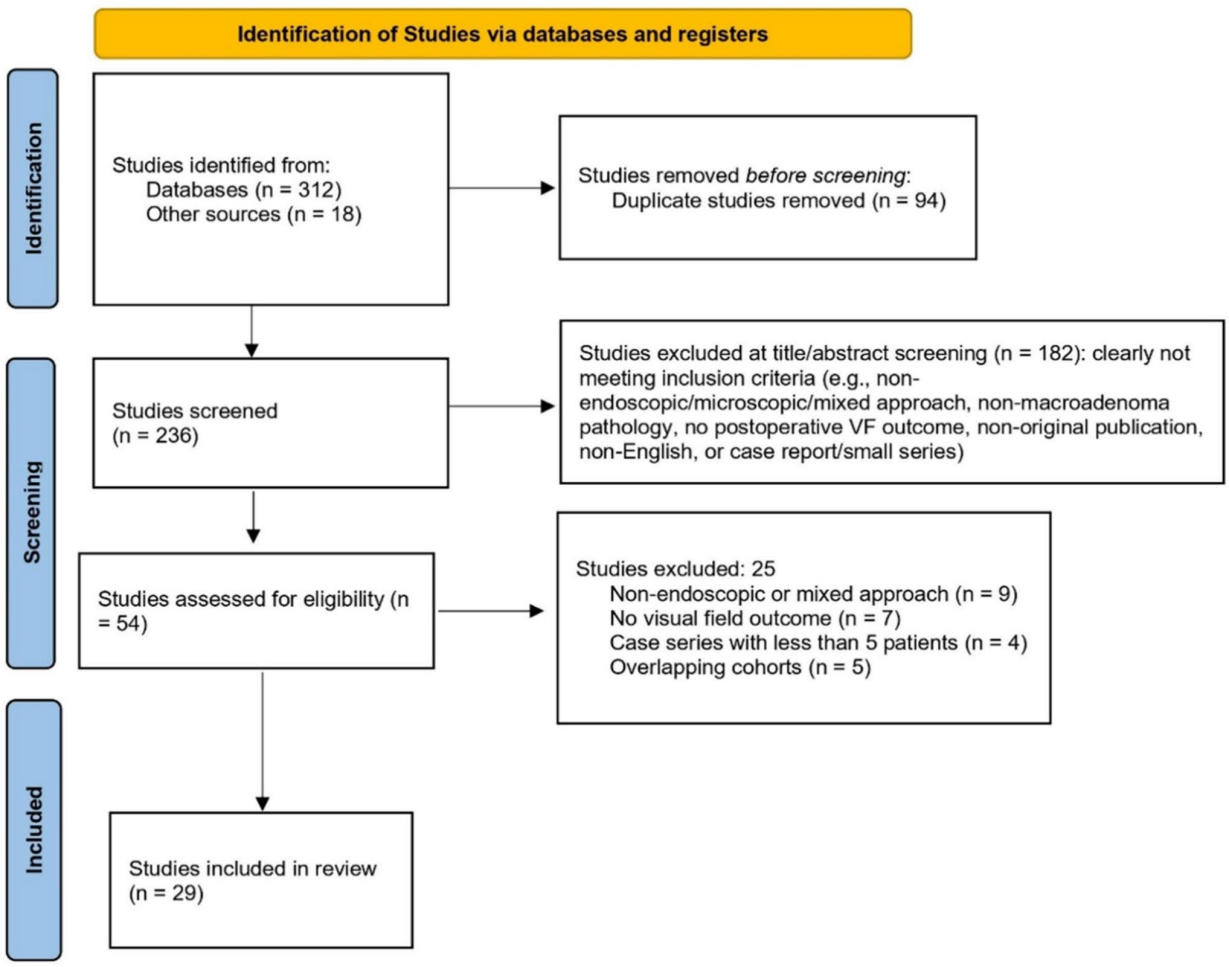
PRISMA 2020 flow diagram for study selection showing identification, screening, eligibility, and inclusion of studies in the qualitative and quantitative synthesis

**Fig. 2:**
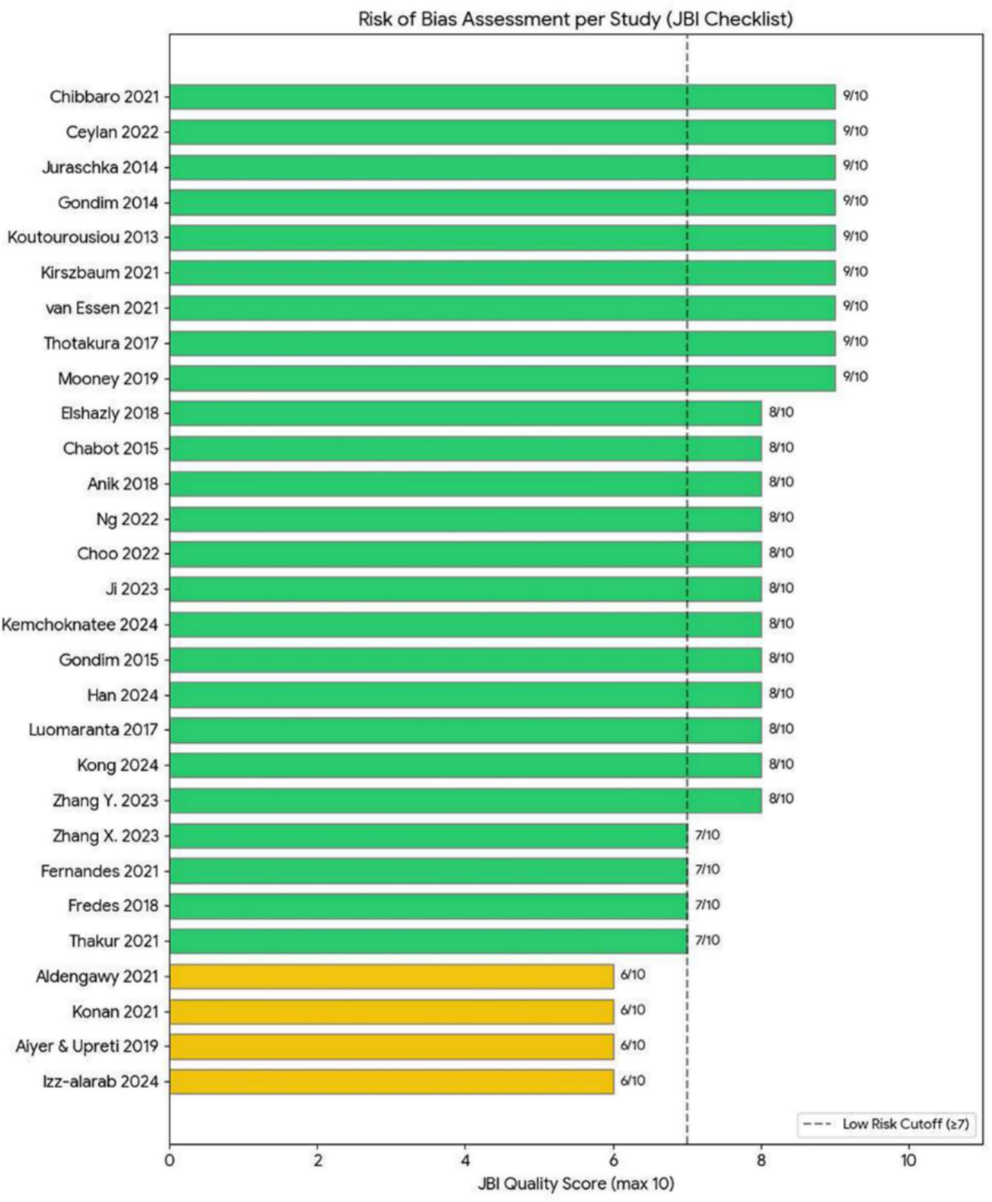
Assessing bias probability among studied sources. Graphical representation of JBI quality scores for all included studies. Dashed line indicates low bias risk threshold (7/10). The bulk of research was classified as low risk of bias, with a minority identified as moderate. This figure is original to this submission so no credit or license is needed

Searches were performed through iterative database queries, and PRISMA flow (Fig. 1) counts were reconstructed based on the final process of eligibility determination and inclusion. Quality evaluation and bias risk assessment were conducted using the Joanna Briggs Institute (JBI) Critical Appraisal Checklist, resulting in a score of 7, which suggests a low probability of bias (Table 1, Fig. 2). Most of the studies exhibited a low risk of bias; a lesser portion were identified as moderate risk. Data collection encompassed study type, sample size, follow-up duration, and outcomes such as VF improvement, normalization, deterioration, and cerebrospinal fluid (CSF) leakage. Pooled proportions were derived using a random-effects model with inverse variance weighting and Freeman-Tukey double arcsine transformation to stabilize variances. Between-study heterogeneity was evaluated using Cochran's Q statistic and quantified with the I^2^ metric, while prediction intervals were computed to approximate the range of potential effects in future similar studies. The systematic review and meta-analysis were registered in PROSPERO [ID # masked for review]. All statistical analysis was conducted on metanlysisonline.com.

**Table 1:** Study quality and risk of bias

Author, year	Study design	JBI score	Risk of bias
Chibbaro et al., 2021 [13]	Prospective	9/10	Low risk
Ceylan et al., 2022 [12]	Retrospective	9/10	Low risk
Juraschka et al., 2014 [30]	Retrospective	9/10	Low risk
Gondim et al., 2014 [22]	Retrospective	9/10	Low risk
Koutourousiou et al., 2013 [36]	Retrospective	9/10	Low risk
Castle-Kirszbaum et al., 2022 [9]	Retrospective	9/10	Low risk
van Essen et al., 2021 [49]	Retrospective	9/10	Low risk
Thotakura et al., 2017 [48]	Prospective	9/10	Low risk
Mooney et al., 2018 [40]	Retrospective	9/10	Low risk
Chabot et al., 2015 [12]	Retrospective	8/10	Low risk
Anik et al., 2018 [5]	Retrospective	8/10	Low risk
Ng et al., 2022 [42]	Retrospective	8/10	Low risk
Choo et al., 2022 [14]	Retrospective	8/10	Low risk
Elshazly et al., 2018 [17]	Retrospective	8/10	Low risk
Ji et al., 2023 [29]	Retrospective	8/10	Low risk
Kemchoknatee et al., 2024 [32]	Retrospective	8/10	Low risk
Gondim et al., 2015 [23]	Retrospective	8/10	Low risk
Han et al., 2024 [25]	Retrospective	8/10	Low risk
Luomaranta et al., 2017 [39]	Retrospective	8/10	Low risk
Kong et al., 2024 [35]	Retrospective	8/10	Low risk
Zhang et al., 2023 [52]	Retrospective	8/10	Low risk
Zhang et al., 2023 [53]	Retrospective	7/10	Low risk
Fernandes et al., 2021 [19]	Retrospective	7/10	Low risk
Fredes et al., 2017 [20]	Retrospective	7/10	Low risk
Thakur et al., 2021 [47]	Retrospective	7/10	Low risk
Konan et al., 2021 [34]	Retrospective	6/10	Moderate risk
Aiyer & Upreti, 2020 [2]	Prospective	6/10	Moderate risk
Aldengawy et al., 2021 [3]	Retrospective	6/10	Moderate risk
Izz-alarab et al., 2024 [28]	Retrospective	6/10	Moderate risk

Inclusion summary: study type, JBI score, bias level. Most studies were retrospective, with key analyses demonstrating low bias potential

## Results

236 studies were screened, of which, 29 met criteria and included 2,943 patients (Table 2, Fig. 1) [2, 3, 5, 9, 11–14, 17, 19, 20, 22, 23, 25, 28–30, 32, 34–36, 39, 40, 42, 47–49, 52, 53]. The unweighted mean age was 51.7 years. Duration of symptoms prior to surgical intervention was inadequately documented in only 9 studies [5, 12, 19, 25, 29, 32, 48, 49, 52] (827 patients),with an unweighted mean duration of 5.9 months, an N-weighted mean of 4.8 months, and a median of 5.4 months. Of these, 3 studies [29, 49, 52] (348/827 patients) indicated median symptom duration < 3 months, 2 studies [5, 25] (235/827) indicated 3–6 months, 3 studies [12, 32, 48] (226/827) indicated 6–12 months, and only 1 study [19] (18/827) indicated 12 months. Follow-up duration was documented across 25 studies involving a total of 2,735 patients, with an unadjusted mean of 24.5 months, a median of 15.0 months, and an N-weighted mean of 26.0 months [2, 3, 5, 9, 11–14, 17, 22, 23, 25, 28–30, 32, 34–36, 40, 47–49, 52, 53]. Follow-up durations ranged from 1.7 to 60 months, with 4 studies [19, 20, 39, 42] (208 patients) lacking quantifiable timeframes. Preoperative VF analysis was conducted in all studies as a criterion for inclusion [2, 3, 5, 9, 11–14, 17, 19, 20, 22, 23, 25, 28–30, 32, 34–36, 39, 40, 42, 47–49, 52, 53].

**Table 2:** Study characteristics

Author, year	Country/Center	Study Design	*N* (Total Patients)	Mean/Median Age	% Female	Symptom Duration (months)	Tumor Size/Volume	Knosp/Hardy/Fujimoto Grade	Pre-op VF Assessment Method	Follow-up Duration
Elshazly et al., 2018 [17]	USA – Thomas Jefferson University Hospital	Retrospective chart review	55	Mean 55.5 (22–88)	36%	Not reported	Mean diameter 5.1 cm; SSE 96%	Knosp 1–4 (69% invasion)	Humphrey VF testing	Mean 41 months (9–95)
Fernandes et al., 2021 [19]	Portugal – Hospital de Egas Moniz, Lisbon	Retrospective observational cross-sectional	18	Mean 57.4 ± 16.5	44.4%	Mean 12.8 ± 14.4	Mean 33.1 ± 9.2 mm	Not reported	Octopus VF; LogMAR VA; OCT RNFL	Not specified
Izz-alarab et al., 2024 [28]	Egypt – Ain Shams University, Nasser Institute	Retrospective cohort	42	Mean 43.6 (22–66)	45.2%	Not specified	27 large, 15 giant	Knosp: 29/42 invaded; 9 grade 4	Humphrey + Snellen	Mean 12.3 months (6–23)
Koutourousiou et al., 2013[36]	USA – University of Pittsburgh	Retrospective review	54	Mean 52.9 (18–80)	15%	Not reported	Mean 50 mm, 32.9 cm^3^	51/54 invasion; Grades 0–4	Ophthalmologic exam	Mean 37.9 months
Gondim et al., 2014 [24]	Brazil – Fortaleza, Campinas, Belo Horizonte	Retrospective review	50	Mean 48.2 (18–78)	34%	Not reported	Mean 5.4 cm	Knosp 2–4 in 16 pts	Ophthalmologic exam	Mean 5 yrs (0.3–13)
Ceylan et al., 2022 [22]	Turkey – Kocaeli University	Retrospective review	205	Mean 46.95 (16–80)	30.2%	Not reported	Mean 46.6 mm (40–87)	87 grade 1–2, 118 grade 3–4	Humphrey VF + Snellen	Mean 50 months (9–247)
Zhang et al., 2023 [52]	China – West China Hospital	Retrospective prognostic imaging study	130	Mean 49.2 ± 13.3	57%	Not reported	Mean 24 mm	Not reported	Octopus/Humphrey automated	12 months
Han et al., 2024 [25]	Pusan National Univ. Yangsan Hospital, Korea	Retrospective	35	Mean 58.3 ± 11.5	60%	~ 4	26 mm; volume predictor	NR	Humphrey analyzer	6 months
Juraschka et al., 2014 [30]	Toronto Western Hospital, Canada	Retrospective	73	Mean 54.5 ± 14.8	32%	NR	Diameter 4.1 cm; volume 18.4 cm^3^	Knosp 0–4 reported	Automated perimetry	3–14 months
Chabot et al., 2015 [12]	Hofstra–North Shore LIJ, Geisinger, USA	Retrospective	39	Mean 56.3 ± 15.6	36%	7	3.4 cm (range 3–6.5)	0–4 reported	Formal perimetry	12 months
Aiyer & Upreti, 2020 [2]	Baroda Medical College & GMC Silvassa, India	Prospective	14	Mean 43.6 (range 19–73)	64%	NR	15–44 mm; mean 28.5 mm	NR	Perimetry	Up to 2 years
Konan et al., 2021 [34]	Yopougon & Bouaké, Côte d’Ivoire	Retrospective	50	46.6 ± 1.7 (range 16–65)	36%	NR	74% macroadenomas; 20% apoplexy	Knosp 0–2: 60%; 3–4: 40%	Computerized campimetry	6 months
Thakur et al., 2021 [47]	USA – Pacific Neuroscience Institute, Saint John’s Cancer Institute (Santa Monica, CA)	Retrospective cohort (prospectively maintained DB, 2010–2020)	514	Mean 51 ± 16 yrs	52.3%	NR	78% macroadenomas; 18 giant; size categories (< 20, 20–29, ≥ 30 mm)	Cavernous sinus invasion (incl. Knosp 4): 121 cases	Formal perimetry/VA in chiasmal compression	Mean 27.4 ± 26.6 mo (min 3)
Mooney et al., 2019 [20] [40]	USA – Multicenter (BNI, PNI, UPMC, OSU, WashU, Northwestern)	Prospective multicenter observational (subset with perimetry)	30 (with both pre/post perimetry)	Mean 62.2 yrs (median 63.5)	33%	NR	Mean max diameter 27.1 mm (9.6–51.9)	Knosp 0–2: 70%; 3–4: 30%. Hardy sellar IV: 37%	Humphrey 30–2 (58%), 24–2 (37%)	Mean 2.4 mo (VF); pre → post interval mean 4.3 mo
Ng et al., 2022 [42]	Hong Kong – Queen Elizabeth Hospital	Retrospective single-center (2015–2018)	108	Mean 59.1 ± 12.9 yrs	40%	NR	Absolute & supracarotid height correlated with VIS	Knosp used; downgrading correlated with VA/VF gains	Standard ophthalmologic perimetry; VIS scale used	NR
Kemchoknatee et al., 2024 [32]	Thailand – Rajavithi Hospital, Bangkok	Retrospective cohort (2016–2020)	87 (174 eyes)	Mean 50 ± 12.8 yrs	64.4%	Median 6 (IQR 1–60); improved 3 vs non-improved 12 (p < 0.001)	79.3% macroadenomas; volume assessed	Hardy A–E; Knosp 0–3 (not predictive)	Humphrey 30–2; reliability < 20% fixation/false responses	Mean 47.5 ± 28.5 mo
van Essen et al., 2021[49]	Netherlands – University Medical Center Utrecht	Retrospective cohort (2010–2015)	100	Mean 53.5 ± 15.7 yrs	38%	Time from ophthalmology → surgery: median 73 days	Macro 89%; mean max diameter 2.6 cm; mean SSE 1.15 cm	Knosp 0–4; Fujimoto 0–4 (39.8% grade 4)	Humphrey/Octopus/Goldmann; ophthalmologist review	Median 52 days (IQR 43–134)
Luomaranta et al., 2017 [39]	Finland – Oulu University Hospital	Retrospective cohort	47	54.7 yrs (18.9–80.1)	55.3%	Not reported	Mean 13.8 mL (VFD) vs 6.2 mL (no VFD); SSE 16.6 vs 6.6 mm	Hardy A 38.3%, B 38.3%, C 14.9%, 0 8.5%	Goldmann 74.5%, Humphrey 6.4%, Both 14.9%, Octopus 2.1%	Not specified
Gondim et al., 2015 [23]	Brazil – General Hospital of Fortaleza	Retrospective cohort	55	72.5 ± 1.9 yrs	54.5%	Not reported	Mean 33 mm; 32.7% cystic	Knosp 2: 5.4%, 3: 9%, 4: 14.5%	Ophthalmologic evaluation	50 months (mean)
Fredes et al., 2017 [20]	Chile – Univ. de Concepción & Brazil (Curitiba)	Cross-sectional (retrospective review)	35	50.2 yrs (26–75)	51.5%	Not reported	All macroadenomas; 80% SSE	Hardy-Wilson II 83.8%, III 8.1%, IV 8.1%	Goldmann (all)	Not specified
Castle-Kirszbaum 2022 [9]	Australia – Monash, St. Vincent’s, Royal Melbourne	Prospective cohort	304	53.8 ± 16 yrs	53%	Not reported	Median volume 6.28 mL (with VFD)	Knosp 0–4; Hardy 0–4, A–E	Humphrey (all)	3 months (acute)
Anik et al., 2018 [5]	Turkey – Kocaeli Univ. & Bakırkoy Dr. Sadi Konuk Hospital	Prospective cohort	200	46.7 ± 11.6 yrs (18–83)	52%	5.4 months	Mean 8,871 mm^3^	Not reported	Humphrey 30–2	3 years (serial)
Aldengawy et al., 2021[3]	Egypt – Al-Azhar Univ. Hospital	Prospective case series	15	40.7 yrs (16–57)	40%	Not reported	All ≥ 4 cm (giant)	Hardy A–E distribution	Goldmann/confrontation	1 year
Thotakura et al., 2017 [48]	India – Nizam’s Institute of Medical Sciences	Prospective series	100	42.5 yrs (14–74)	48%	11.8 months; < 1 yr predicted better	Mean 32.97 mm; SSE 14.95 mm	Parasellar ext. 27 pts (Knosp 1–4)	VIS (acuity + field); Goldman/clinical	43.5 months
Ji et al., 2023 [29]	China – The First Affiliated Hospital of Soochow University, Suzhou	Retrospective, single-center	28	45.1 ± 14.7 years	64.3%	Median 2.5 (IQR 0–12)	Median diameter 20 mm (IQR 15–29.25)	Not reported	Octopus 900 static perimetry (G-TOP)	≤ 15 months
Zhang et al., 2023 [53]	China – West China Hospital, Sichuan Univ + multicenter validation	Prospective cohort + external validation	220	~ 47 years (46 vs 50 recovery/non)	53.2%	Median 1.0 (IQR 2.0–2.5)	Max diameter ~ 24–26 mm; SSE 15–17 mm	Not specified	Humphrey Field Analyzer	6 months
Chibbaro et al., 2021 [13]	France, Italy, UK – 5 centers	Prospective multicenter cohort	96	52.2 years (26–81)	42.7%	Not specified	Mean CC diameter 46.5 mm	Knosp 0–4 distribution	Ophthalmological exam + OCT	52.4 months (24–88)
Choo et al., 2022 [14]	Republic of Korea – Yeungnam University Hospital	Retrospective cohort (20 years)	181	54 (21–79)	47.0%	Not specified	84.5% macro, 12.7% giant, 2.8% micro	Hardy 0–4; Knosp 0–4	Automated perimetry	Median 58 mo (6–255)
Kong et al., 2024 [35]	China – First Affiliated Hospital of Harbin Medical University	Retrospective comparative cohort	58	50.8 ± 12.7	72.4%	Not reported	24.5 mm	Hardy used; Knosp < 3	Ophthalmologic perimetry	3 months

The majority of studies incorporated automated perimetry [3, 5, 9, 11, 12, 14, 17, 19, 20, 25, 28–30, 32, 34, 39, 40, 42, 47–49, 52, 53] (23 studies, 79%), specifically Humphrey [11, 17, 25, 28, 52, 53] (n = 12, 41%), Octopus [5, 9, 19, 39, 49, 52, 53] (n = 5, 17%) and Goldmann [3, 20, 39, 48, 49] (n = 5, 17%) VF assessments. Six studies [2, 13, 22, 23, 35, 36] employed unspecified ophthalmologic perimetry, and 1utilized confrontation perimetry exclusively [3]. Baseline VF severity was variably documented. Eight studies [9, 25, 29, 32, 42, 48, 52, 53] included quantitative data (e.g., mean deviation, MD) indicating moderate-severe impairment, while the remaining 21 studies [2, 3, 9, 11–14, 17, 19, 20, 22, 23, 28, 30, 34–36, 39, 40, 47, 49] provided qualitative descriptions (e.g., bitemporal hemianopia, generalized depression), precluding detailed severity analysis.

Tumor size and volume were variably recorded. Maximum tumor diameter was reported in 17 studies [2, 12, 13, 17, 19, 22, 23, 25, 29, 30, 35, 36, 40, 48, 49, 52, 53] (1,140 patients), with an unweighted mean of 35.2 mm, an N-weighted mean of 36.9 mm, and a range of 20.0–54.0 mm. Volume reporting in 5 studies [5, 9, 30, 36, 39] (678 patients): 6.3–32.9 mL (unweighted mean 16.1 mL; N-weighted mean 11.0 mL). Invasion grading was reported in only 12 studies [17, 20, 22, 23, 28, 30, 32, 34, 39, 40, 47, 49] (41%) using Knosp, Hardy, or Fujimoto systems, and in Knosp-reporting cohorts, the median proportion of patients with high-grade invasion (Knosp 3–4) was 35% (range 23.5% to 57.6%). In general, tumor invasion or chiasmal extension grading of any type was reported across 22 studies [3, 9, 11–14, 17, 20, 22, 23, 28, 30, 32, 34–36, 39, 40, 42, 47–49], with Knosp (16 studies) [9, 12–14, 17, 22, 23, 28, 32, 34, 35, 40, 42, 47–49] and Hardy (8 studies) [3, 9, 14, 20, 32, 35, 39, 40] being the most utilized systems; whereas 7 studies [19, 25, 52, 53] lacked grading data entirely. Of the studies with quantitative distributions, approximately 1/4 to 3/5 of tumors were high grade, but heterogeneity precluded pooled analysis.

**Fig. 3:**
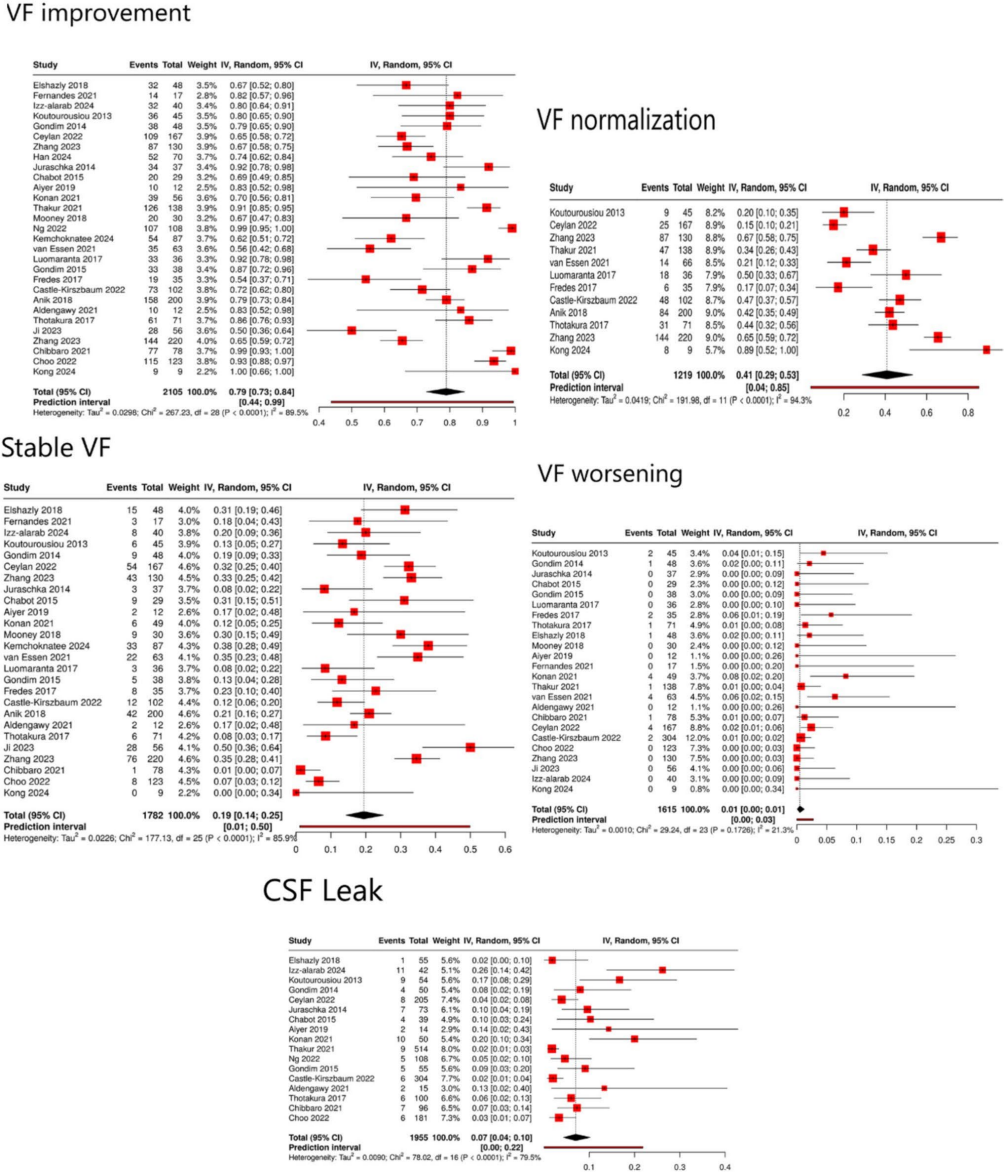
Pooled postoperative outcomes after endoscopic endonasal transsphenoidal surgery for pituitary macroadenomas (random-effects model). **A** Pooled proportion of patients experiencing postoperative VF improvement following endoscopic transsphenoidal surgery. Random effects estimate: 0.79 (95% CI: 0.73–0.84); I^2^ = 89.5% indicating significant heterogeneity. **B** Aggregated proportion of patients experiencing VF normalization post-surgery; random effects estimate 0.41 (95% CI: 0.29–0.53), with significant heterogeneity (I^2^ = 94.3%). **C** Pooled proportion of patients exhibiting stable visual fields (meaningless change) following surgery; random effects estimate 0.19 (95% CI: 0.14–0.25), with notable heterogeneity (I^2^ = 85.9%). **D** Combined proportion of patients experiencing postoperative VF deterioration; random effects estimate 0.01 (95% CI: 0.00–0.01), with minimal heterogeneity (I^2^ = 21.3%). **E** Combined postoperative CSF leak rate; random effects estimate 0.07 (95% CI: 0.04–0.10), with high heterogeneity (I^2^ = 79.5%). This figure is original to this submission so no credit or license is needed

In total, 29 studies included 2,943 patients who underwent EETS removal of pituitary macroadenomas, with visual outcomes uniformly favorable across all reported categories. (Table 3) [2, 3, 5, 9, 11–14, 17, 19, 20, 22, 23, 25, 28–30, 32, 34–36, 39, 40, 42, 47–49, 52, 53]. VF improvement was observed in 2,105 patients across 29 studies [2, 3, 5, 9, 11–14, 17, 19, 20, 22, 23, 25, 28–30, 32, 34–36, 39, 40, 42, 47–49, 52, 53], resulting in a pooled improvement rate of 79% (95 CI: 73–84%; prediction interval 44–99%) (Fig. 3); whereas normalization was achieved in 1,219 patients across 12 studies [5, 9, 11, 20, 35, 36, 39, 47–49, 52, 53] with a pooled rate of 41% (95% CI: 29–53%; prediction interval 4–85%) (Fig. 3). Stable VF was reported in an estimated 19% (95% CI: 14- 25%) of 1,782 patients [2, 3, 5, 9, 11, 12, 14, 17, 19, 20, 22, 23, 28–30, 32, 34–36, 39, 40, 48, 49, 52, 53]; whereas postprocedural deterioration was observed in only 1% (95% CI: 0–1%; prediction interval:0–3%) of 1,615 patients [2, 3, 9, 11–14, 17, 19, 20, 22, 23, 28–30, 34–36, 39, 40, 47–49, 52]. Complications were varied in reporting; however, one consistently reported outcome was postoperative cerebrospinal fluid leak CSF. A total of 1,955 patients across 17 studies [2, 3, 9, 11–14, 17, 22, 23, 28, 30, 34, 36, 42, 47, 48] demonstrated a pooled CSF leak rate of 7% (95% CI: 4–10%; prediction interval 0–22%).

**Table 3:** Visual field and visual acuity outcomes

Author, year	VF improvement (*n*, %)	VF normalization (*n*, %)	Stable VF (*n*, %)	Worsened VF (*n*, %)	Visual acuity outcomes
Elshazly et al., 2018 [17]	32/48 (66%)	Not separated	15/48 (31%)	1/48 (2%)	Not separately reported
Fernandes et al., 2021 [19]	14/17 (82.4%)	Not reported	3/17 (17.6%)	0	Improved LogMAR VA
Izz-alarab et al., 2024 [28]	32/40 (80%)	Not reported	8/40 (20%)	0	Included
Koutourousiou et al., 2013 [36]	36/45 (80%)	9/45 (20%)	6/45 (13.3%)	2/45 (4.4%)	Not separate
Gondim et al., 2014 [24]	38/48 (76%)	Not reported	9/48 (18.7%)	1/48 (2.1%)	Not separate
Ceylan et al., 2022 [22]	109/167 (65.3%)	25/167 (15%)	54/167 (32.3%)	4/167 (2.4%)	Not separate
Zhang et al., 2023 [52]	87/130 (66.9%)	87/130 (66.9%)	43/130 (33.1%)	0	Not separate
Han et al., 2024 [25]	26/30 thick RNFL; 26/40 thin RNFL improved/stable	NR	Included with improved	1 eye	Slight BCVA improvement (NS)
Juraschka et al., 2014 [30]	34/37 (92%)	NR	3 (8%)	0	73% improved, 5% worsened
Chabot et al., 2015 [12]	20 (69%)	NR	9 (31%)	0	65% improved, none worsened
Aiyer & Upreti, 2020 [2]	10/12 (83%)	NR	2/12 (17%)	0	NR
Konan et al., 2021 [34]	39 (70%)	NR	6 (11%)	4 (7%)	55% had VA loss; partial recovery not clearly separated
Thakur et al., 2021 [47]	126/138 (91.3%)	≈34.1% (~ 47/138)	NR	0 permanent; 1 transient	No permanent worsening
Mooney et al., 2019 [40]	20/30 (67%) rater1; 18/30 (60%) rater2	NR	7/30 (23%) r1; 9/30 (30%) r2	0%	NR
Ng et al., 2022 [42]	≈99% improved	NR	NR	NR	88% improved
Kemchoknatee et al., 2024 [32]	62.1% improved at 1 year (+ 2.4 dB mean)	NR	37.9%	None reported	Mean logMAR − 0.56 at 1 yr (significant)
van Essen et al., 2021[49]	35/63 (55.5%)	14/66 (21.2%)	22/63 (34.9%)	4/63 (6.3%)	Mean VA 0.67 → 0.84 (p = 0.04)
Luomaranta et al., 2017 [39]	33/36 (91.7%)	18/36 (50%)	3/36 (8.3%)	0	VA improved in 71%
Gondim et al., 2015 [23]	33/38 (86.8%)	Not reported	5/38 (13.2%)	0	Not detailed
Fredes et al., 2017 [20]	19/35 (54.2%)	6/35 (17.2%)	8/35 (22.8%)	2/35 (5.8%)	Not reported
Castle-Kirszbaum 2022 [9]	73/102 (71.6%)	48/102 (16.9%)	12/102 (11.7%)	2/304 (0.7%)	Not emphasized
Anik et al., 2018 [5]	158/200 (79%)	84/200 (42%)	42/200 (21%)	Not specified	Improved significantly
Aldengawy et al., 2021[3]	10/12 (83.3%)	Not reported	2/12 (16.7%)	0	83% improved
Thotakura et al., 2017 [48]	61/71 (85.9%)	31/71 (43.7%)	6/71 (8.4%)	1/71 (1.4%)	Integrated in VIS
Ji et al., 2023 [29]	28/56 eyes (50%)	Not reported	28/56 eyes (50%)	0	Not reported
Zhang et al., 2023 [53]	144/220 (65.5%)	144/220 (65.5%)	76/220 (34.5%)	Not reported	Not reported
Chibbaro et al., 2021 [13]	77/78 (98.7%)	Not specified	1/78 (1.3%)	1 monocular blindness	Improved in 77/78
Choo et al., 2022 [14]	115/123 (93.5%)	Not reported	8/123 (6.5%)	0	Included in impairment
Kong et al., 2024 [35]	9/9 (100%)	8/9 (88.9%)	0	0	Improved with VF

## Discussion

The current single-arm meta-analysis further supports EETS as a highly effective and safe intervention for improvement of VF deficits, secondary to pituitary macroadenoma [4, 6, 19, 20, 24, 29, 41, 44, 45]. Our study showed that EETS reliably resulted in substantial VF improvement with minimal postoperative deterioration, corroborating and expanding upon previous institutional experiences and meta-analyses of visual outcomes following endoscopic pituitary procedures [19, 20, 29, 41, 49]. Visual improvement was seen in a weighted pooled average of 79% of patients.

The process of visual improvement subsequent to chiasmal decompression is optimally understood as a multiphasic phenomenon. Early improvement within days to weeks is believed to reflect rapid reversal of conduction block and re-establishment of axoplasmic transport in chronically compressed fibers [21, 33, 41, 43]. The subsequent gradual gains over months to years are likely related to remyelination, reorganization of synaptic connections, and reestablishment of optimal retinal and post-chiasmal circuitry, as supported by optical coherence tomography (OCT) and electrophysiologic studies showing progressive improvements in retinal nerve fiber layer (RNFL) and visual pathway conduction following surgery [33, 43]. These findings collectively provide evidence that EETS not only halts further deterioration but also facilitates significant biological recovery when a sufficient number of axons remain viable.

Duration of symptoms is identified as a critical factor influencing reversibility. Gnanalingham et al. indicated that patients who undergo surgery within approximately 6 months of visual decline exhibit a significantly increased probability of near-normal postoperative visual fields [21]; whereas delayed intervention exceeding 1 year often results in residual deficits despite sufficient decompression [21, 38]. In our systematic review, the majority of patients were identified within a relatively brief timeframe following symptom presentation, which may have contributed to the observed consistent favorable visual recovery. These observations emphasize the necessity of prompt referral and surgical management upon detection of VF compromise [21, 29, 38].

Tumor size, suprasellar extension, and cavernous sinus invasion are additional factors influencing results. Larger and more invasive macroadenomas are associated with worse initial vision and a higher likelihood of residual tumor, but they can also demonstrate significant absolute improvement once severe chiasmal compression is alleviated [26, 29, 38, 46, 49]. Despite more complex presentation, improved visual function can still be seen [26, 49]. Analysis of adenoma dimensions and degree of suprasellar extension on preoperative VF loss indicates that vertical compression appears to be the strongest predictor of chiasmal dysfunction, rather than purely intrasellar volume [26, 49]. Even in cohorts with identified Knosp 3–4 invasion, endoscopic visualization of the opticocarotid region allows for precise decompression while minimizing damage to neurovascular structures, which can help maintain low rates of new visual deficits [6, 20, 29, 44, 45].

Initial VF severity and anterior visual pathway integrity are equally strong predictors. Numerical data suggest that superior preoperative MD and thicker RNFL correlate with improved postoperative results, while severe preoperative loss and optic atrophy serve as negative predictors for full recovery [21, 29, 38, 43, 46]. The pattern of early postoperative improvement notably initiation within the inferior temporal field may indicate selective recovery of residual fibers [29]. Such findings underscore the importance of integrating automated perimetry and structural imaging into standardized protocols for future research, enhancing predictive accuracy [21, 29, 38, 43, 46, 49].

From a safety perspective, the minimal rate of postoperative visual deterioration reported in contemporary EETS series is comparable to modern microsurgical and transcranial approaches, while older series may have had higher rates of new or worsened visual deficits due to brain retraction and limited visualization [4, 6, 19, 24, 44, 45]. CSF leak remains the most clinically significant postoperative complication. Modern endoscopic series of pituitary and skull base tumors today have reported postoperative CSF leaks at an overall rate of between 2–8%, dependent on patient, tumor and reconstructive factors [10, 31, 37, 50, 51]. The introduction of vascularized nasoseptal flaps and multilayer reconstruction techniques has significantly reduced leak rates, especially in high-risk skull-base defects [8]. Additionally, surgical proficiency may improve over time, suggesting further enhancement of visual outcomes and complication profile over surgical careers [1, 7, 27, 31, 44].

### Limitations

This meta-analysis is limited by the predominantly retrospective nature of included studies and by non-standardized VF assessment and reporting. Perimetry platforms (Humphrey/Octopus/Goldmann), reporting formats (qualitative vs quantitative), follow-up duration, and definitions of “improvement” versus “normalization” varied substantially across cohorts. Accordingly, we pooled harmonized categorical outcomes (improved/normalized/stable/worsened) using a random-effects framework and reported prediction intervals to contextualize heterogeneity. These results therefore provide benchmark outcome rates after EETS rather than definitive conclusions regarding recovery time-course or prognostic predictors. Future prospective studies and registries using uniform automated perimetry metrics (e.g., MD and VFI), standardized OCT/RNFL reporting, and consistent tumor grading (Knosp/Hardy) are needed to enable stronger inference regarding visual recovery determinants and timing.

## Conclusion

In summary, the cumulative data suggest that EETS for pituitary macroadenomas offers a favorable likelihood of significant VF improvement, coupled with minimal morbidity and a controlled CSF leak rate. When combined with early intervention, precise endoscopic execution, and modern skull base reconstruction, such data advocate for EETS as the optimal method for addressing chiasmal compression caused by pituitary macroadenomas. These pooled figures serve as benchmark outcome rates to inform patient counseling, pending prospective studies using standardized perimetry metrics.

## Data Availability

This review analyzed published data, which are available in PROSPERO. Additional synthesized data are available from the corresponding author on reasonable request.

## Abbreviations

CSF: Cerebrospinal fluid
EETS: Endoscopic endonasal transsphenoidal surgery
PRISMA: Preferred reporting items for systematic reviews and meta-analyses
VF: Visual field
